# Plastid nucleoids: evolutionary reconstruction of a DNA/protein structure with prokaryotic ancestry

**DOI:** 10.3389/fpls.2015.00220

**Published:** 2015-04-08

**Authors:** Jeannette Pfalz, Thomas Pfannschmidt

**Affiliations:** ^1^Department of Plant Physiology, Institute of General Botany and Plant Physiology, Friedrich-Schiller-University JenaJena, Germany; ^2^UMR5168, University Grenoble-AlpesGrenoble, France; ^3^Centre National de la Recherche Scientifique, UMR5168Grenoble, France; ^4^Commissariat à l'Energie Atomique et aux Energies Alternatives, iRTSV, Laboratoire de Physiologie Cellulaire and VégétaleGrenoble, France; ^5^Institut National de la Recherche Agronomique, USC1359Grenoble, France

**Keywords:** plastids, nucleoids, endosymbiosis, replication, transcription, post-transcriptional events

Understanding the evolutionary establishment of plastids within eukaryotic cells and the principles that govern the process of endosymbiosis have been integral to research in plant sciences during the past three decades. Determination of the primary DNA sequence of the plastome from many plants and algae represented a milestone in this field, making it possible to deduce evolutionary lineages via bioinformatic approaches. These have greatly improved our understanding of endosymbiosis, the evolution of plastids and the reshaping of the eukaryotic host genome following massive horizontal gene transfer from the ancient cyanobacterial progenitor toward the host nucleus. Astonishingly, much less is known about the current structure and organization of plastid DNA and its association with different kinds of proteins that are involved in its stabilization, replication and expression.

As in bacteria, the DNA in plant and algal plastids appears to be organized in nucleoids that can be easily visualized by fluorescence microscopy using DNA-specific dyes. This approach identifies nucleoids as dots of distinctive shape that are located close to the thylakoid or envelope membrane depending on the developmental stage of the plastid. However, at the molecular level nucleoids represent a less well defined structure as they have been found to be a highly dynamic protein/DNA/RNA structure. In particular, its protein subunit composition is highly variable depending on the developmental stage of the plastid and the tissue context in which it resides, as well as on the environmental condition of the organism. In addition, the structure and organization of the DNA itself is still under debate. A definition of what precisely is a nucleoid in terms of protein subunit composition and structure, therefore, appears to be difficult on the basis of current knowledge. This research topic gives a snapshot of the current state-of-the-art on nucleoids focussing on their structure and composition. It zooms through the different levels of proteins involved in processes that are prerequisite for proper nucleoid structure and faithful gene expression.

The primary topic of the articles in this research topic is the various proteins found in nucleoids or likely associated with them based on their functional contribution to gene expression. Current knowledge and open questions about the organization of nucleoids are summarized in an initial review by Powikrowska et al. (2014). This article discusses the various appearances of nucleoids in different microscopy techniques, focussing heavily on the structural organization of DNA and the proteins that mediate it. It summarizes the characteristics of known plastid nucleoid associated proteins (ptNAPs) proposed to be involved in shaping and organization of nucleoids in plants. It also compares nucleoid morphology and organization in bacteria with that found in plants and extensively discusses the dynamics of nucleoid re-organization during the different phases of chloroplast development. This review is complemented by a research article that analyses the role of the protein Whirly1 in barley (Krupinska et al., 2014). Down-regulation of Whirly1 via RNAi results in the occurrence of larger and more irregularly formed patches of DNA than are normally found in nucleoids. The data suggest an important role for Whirly1 in compacting nucleoid DNA and thereby affecting DNA replication.

These two articles set the scene for a detailed review about the enzymes involved in organellar replication contributed by Moriyama and Sato (2014), who describe the history of studies on organellar DNA polymerases and their enzymatic characteristics, including sensitivity to inhibitors or exonuclease activity. The article furthermore highlights other enzymes involved in replication such as helicases, DNA primase and topoisomerase as well as single-stranded DNA binding proteins. The review also covers the evolution of all these enzymes and their phylogenetic origins and relationships, and ends with an interesting model for the exchange of organellar replication enzymes during the evolution of photosynthetic eukaryotes.

The first level of gene expression is the transcription of the genetic information encoded by DNA. In chloroplasts, RNA is synthesized by two different types of RNA polymerases, the plastid-encoded RNA polymerase (PEP) and nuclear-encoded RNA polymerase (NEP). The PEP enzyme constitutes a genetically chimeric multi-protein complex with plastid-encoded core subunits structurally related to the bacterial *E. coli* RNA polymerase. One new feature of the PEP in higher plants, however, is its assembly with numerous nucleus-encoded eukaryotic components (PEP-associated proteins), which are reviewed in two articles (Yu et al., 2014; Yagi and Shiina, 2014). During the past decade, several approaches have established an im-portant role for such PEP-associated proteins (PAPs) in a variety of biological processes. These include transcriptional regulation, DNA/RNA metabolism, posttranslational modification and detoxification. More recently, it has been proposed that these proteins serve also as building blocks in the PEP assembly, but how exactly these proteins contribute to transcription and gene regulation awaits further investigation.

One important characteristic of plastid gene expression is the observation that PEP activity changes both in a developmentally regulated fashion and in response to environmental variables. Key proteins that mediate these changes in transcription are the different members of the sigma family (e.g., six in *Arabidopsis*) which initiate transcription in a complementary and flexible manner. Their concerted action allow greater flexibility in developmental- and tissue-specific cellular responses (Bock et al., 2014). Other proteins that appear to influence developmental changes of plastid transcription are PRIN2 in *Arabidopsis* (Kremnev and Strand, 2014) or NUS1 in rice (Kusumi and Iba, 2014). PRIN2 was found to generate complexes with another protein called CSP41b (see also below). This complex appears to possess DNA binding activity *in vitro*, suggesting a regulatory role in plastid gene expression (Kremnev and Strand, 2014). NUS1 appears to be a regulator of plastid 16S rRNA expression that is responsible for the establishment of the plastid gene expression machinery in early stages of chloroplast development of rice exposed to low-temperature conditions. It works in conjunction with regulators of organellar and cytosolic nucleotide metabolism, indicating that nucleotide metabolism is essential for chloroplast development (Kusumi and Iba, 2014).

Post-transcriptional regulation is a further important level of control in plastids, and is high-lighted by two opinion articles in this issue (Bohne, 2014; Leister, 2014). The first discusses the roles of rRNA processing and maturation in nucleoids (Bohne, 2014). Based on experimental observations in bacteria, plastids and mitochondria, a new model was developed in which, in organelles, rRNA processing and ribosome assembly most likely take place in nucleoids (Bohne, 2014). The second article focusses on the roles of the CSP41 proteins (e.g., CSP41a and CSP41b) (Leister, 2014). These are multifunctional proteins of high abundance which have been found in several stromal protein complexes in different contexts, including RNA cleavage, RNA stabilization, transcription and carbon metabolism. Considering the abundance, CSP41 may have a key role in RNA stabilization.

The issue closes with a research article which describes an effective biochemical purification strategy that helps to isolate many of the aforementioned proteins from chloroplast nucleoids (Schröter et al., 2014). This strategy might be helpful in future in order to study native properties of nucleoid proteins isolated from plants in different developmental or environmental conditions. In summary, this research topic covers the full breadth of structural and functional implications of plastid nucleoids as currently known. It provides a comprehensive overview to the interested newcomer to the field and demonstrates open questions and topics which promise fundamental new discoveries in the years to come.

## Conflict of interest statement

The authors declare that the research was conducted in the absence of any commercial or financial relationships that could be construed as a potential conflict of interest.

## References

[B1] Bock S. Ortelt J. Link G. (2014). AtSIG6 and other members of the sigma gene family jointly but differentially determine plastid target gene expression in *Arabidopsis thaliana*. Front. Plant Sci. 5:667. 10.3389/fpls.2014.00667 25505479 PMC4243499

[B2] Bohne A. V. (2014). The nucleoid as a site of rRNA processing and ribosome assembly. Front. Plant Sci. 5:257. 10.3389/fpls.2014.00257 24926303 PMC4046486

[B3] Kremnev D. Strand A. (2014). Plastid encoded RNA polymerase activity and expression of photosynthesis genes required for embryo and seed development in *Arabidopsis*. Front. Plant Sci. 5:385. 10.3389/fpls.2014.00385 25161659 PMC4130184

[B4] Krupinska K. Oetke S. Desel C. Mulisch M. Schäfer A. Hollmann J.. (2014). WHIRLY1 is a major organizer of chloroplast nucleoids. Front. Plant Sci. 5:432. 10.3389/fpls.2014.00432 25237316 PMC4154442

[B5] Kusumi K. Iba K. (2014). Establishment of the chloroplast genetic system in rice during early leaf development and at low temperatures. Front. Plant Sci. 5:386. 10.3389/fpls.2014.00386 25157260 PMC4127815

[B6] Leister D. (2014). Complex(iti)es of the ubiquitous RNA-binding CSP41 proteins. Front. Plant Sci. 5:255. 10.3389/fpls.2014.00255 24936205 PMC4047790

[B7] Moriyama T. Sato N. (2014). Enzymes involved in organellar DNA replication in photosynthetic eukaryotes. Front. Plant Sci. 5:480. 10.3389/fpls.2014.00480 25278952 PMC4166229

[B8] Powikrowska M. Oetke S. Jensen P. E. Krupinska K. (2014). Dynamic composition, shaping and organization of plastid nucleoids. Front. Plant Sci. 5:424. 10.3389/fpls.2014.00424 25237313 PMC4154389

[B9] Schröter Y. Steiner S. Weisheit W. Mittag M. Pfannschmidt T. (2014). A purification strategy for analysis of the DNA/RNA-associated sub-proteome from chloroplasts of mustard cotyledons. Front. Plant Sci. 5:557. 10.3389/fpls.2014.00557 25400643 PMC4212876

[B10] Yagi Y. Shiina T. (2014). Recent advances in the study of chloroplast gene expression and its evolution. Front. Plant Sci. 5:61. 10.3389/fpls.2014.00061 24611069 PMC3933795

[B11] Yu Q. B. Huang C. Yang Z. N. (2014). Nuclear-encoded factors associated with the chloroplast transcription machinery of higher plants. Front. Plant Sci. 5:316. 10.3389/fpls.2014.00316 25071799 PMC4080259

